# Supplementary material for: Content-rich biological network constructed by mining PubMed abstracts
Source: BMC Bioinformatics. 2004 Oct 8;5:147. doi: 10.1186/1471-2105-5-147 (PMC528731; doi:10.1186/1471-2105-5-147)
Supplement: Additional File 2 — The original results of the above study (non-essential files are deleted to keep the file size under the limit set by BMC bioinformatics). [file 1471-2105-5-147-S2.bz2 › chilibotAdditionalFile2/dip40/199ID8652557E688/html/ERCC3_ERCC5.html]

 


 **ERCC3** and **ERCC5** 
  
Found 46 abstracts in PubMed, retrieved 40.  
 

 What does Google say? 
 PDF only 
| .edu only 

---

**Interactive relationship** (e.g. stimulation, inhibition, etc)

**Neutral relationship**- Strong functional interactions of TFIIH  [ **ERCC3** ]  with XPC and XPG  [ **ERCC5** ]  in human DNA nucleotide excision repair, without a preassembled repairosome.  Ref: 11259578 Mol Cell Biol, 2001
- Interactions involving the human RNA polymerase II transcription nucleotide excision repair complex TFIIH  [ **ERCC3** ] , the nucleotide excision repair protein XPG  [ **ERCC5** ] , and Cockayne syndrome group B CSB protein.  Ref: 8652557 Biochemistry, 1996
- Novel functional interactions between nucleotide excision DNA repair proteins influencing the enzymatic activities of TFIIH  [ **ERCC3** ] , XPG  [ **ERCC5** ] , and ERCC1 XPF.  Ref: 11141066 Biochemistry, 2001
- XPG  [ **ERCC5** ]  protein interacts with multiple subunits of TFIIH  [ **ERCC3** ]  and with CSB protein.  Ref: 8652557 Biochemistry, 1996
- At physiological ionic strength, TFIIH  [ **ERCC3** ]  associated with significant amounts of XPC HR23B and XPG  [ **ERCC5** ]  but not other repair factors.  Ref: 11259578 Mol Cell Biol, 2001
- To identify the forms of TFIIH  [ **ERCC3** ]  that can function in repair, recombinant XPA, RPA, XPC hHR23B, XPG  [ **ERCC5** ] , and ERCC1 XPF were combined with TFIIH  [ **ERCC3** ]  fractions purified from HeLa cells.  Ref: 10673506 Genes Dev, 2000
- Furthermore, we reveal that TFIIH  [ **ERCC3** ]  inhibits the structure specific endonuclease activities of both XPG  [ **ERCC5** ]  and ERCC1 XPF, responsible for the 3 and 5 incision in NER, respectively.  Ref: 11141066 Biochemistry, 2001

**Non-interactive relationship** (e.g. studied together, co-existance, homology, etc.)

- Defects in the XPB  [ **ERCC3** ] , XPD, and XPG  [ **ERCC5** ]  genes can result in three different syndromes, xeroderma pigmentosum, Cockayne syndrome, or trichothiodystrophy, depending on the specific mutation involved.  Ref: 9425594 Curr Opin Pediatr, 1997
- A set of proteins required for repair of most lesions is RPA, XPA, TFIIH  [ **ERCC3** ] , XPC hHR23B, XPG  [ **ERCC5** ] , and ERCC1 XPF, but additional components have not been excluded.  Ref: 10673506 Genes Dev, 2000
- Cell lines defective in other genes involved in NER XPB  [ **ERCC3** ] , XPD, and XPG  [ **ERCC5** ] , together with a mutant defective in nonhomologous end joining XRCC5, showed only mild sensitivity.  Ref: 11027268 Mol Cell Biol, 2000
- CONCLUSIONS Reduced expression of ERCC1, XPB  [ **ERCC3** ]   **ERCC3** , XPG  [ **ERCC5** ]   **ERCC5** , and CSB ERCC6 is associated with a more than two fold increased risk of SCCHN.  Ref: 11900225 Cancer, 2002
- In contrast to cells bearing mutations in other components of the nucleotide excision repair apparatus XPB  [ **ERCC3** ] , XPD, XPG  [ **ERCC5** ]  and CSB, cells defective for the ERCC1 XPF structure specific nuclease are highly sensitive to cisplatin.  Ref: 12202770 Nucleic Acids Res, 2002
- RESULTS The levels of ERCC1, XPB  [ **ERCC3** ]   **ERCC3** , XPG  [ **ERCC5** ]   **ERCC5** , and CSB ERCC6 transcripts were lower in cases than in controls.  Ref: 11900225 Cancer, 2002
- Human nucleotide excision repair is initiated by six repair factors XPA, RPA, XPC HR23B, TFIIH  [ **ERCC3** ] , XPF ERCC1, and XPG  [ **ERCC5** ]  which sequentially assemble at sites of DNA damage and effect excision of damage containing oligonucleotides.  Ref: 12138203 Mol Cell Biol, 2002
- In each of these cell lines, the genes examined included ERCC1, XPA, XPB  [ **ERCC3** ] , XPD, XPG  [ **ERCC5** ] , CSB and p53.  Ref: 10738106 Cancer Lett, 2000
- Furthermore, reconstitution of the human excision nuclease was observed with a mixture of five repair factors XPA, XPC, XPG  [ **ERCC5** ] , TFIIH  [ **ERCC3** ] , and RPA and the recombinant XPF ERCC1, thus verifying that no additional protein factors are needed for the specific dual incisions characteristic of human excision repair.  Ref: 9013642 J Biol Chem, 1997
- In mammalian cells, the core factors involved in the damage recognition and incision steps of DNA nucleotide excision repair are XPA, TFIIH  [ **ERCC3** ]  complex, XPC HR23B, replication protein A RPA, XPG  [ **ERCC5** ] , and ERCC1 XPF.  Ref: 11259578 Mol Cell Biol, 2001
- These results point toward additional roles for TFIIH  [ **ERCC3** ]  and ATP during NER distinct from a requirement for DNA unwinding in the regulation of the endonuclease activities of XPG  [ **ERCC5** ]  and ERCC1 XPF.  Ref: 11141066 Biochemistry, 2001
- RESULTS Mammalian cell lines XPB  [ **ERCC3** ] , XPD, XPF, XPG  [ **ERCC5** ] , and ERCC1 deficient in the nucleotide excision repair NER gene products show resistance to Et 743.  Ref: 11731295 Chem Biol, 2001
- The dual incision reaction was reconstituted using the purified mammalian proteins XPA, RPA, XPC, TFIIH  [ **ERCC3** ] , XPG  [ **ERCC5** ] , and a fraction containing ERCC1 XPF and IF7.  Ref: 8636155 J Biol Chem, 1996
- Here, we provide evidence that CSB is found not only in the nucleoplasm but also in the nucleolus within a complex CSB IP 150 that contains RNA pol I, TFIIH  [ **ERCC3** ] , and XPG  [ **ERCC5** ]  and promotes efficient rRNA synthesis.  Ref: 12419226 Mol Cell, 2002
- P< ; 10 11 while XPA, XPB  [ **ERCC3** ] , XPC, XPG  [ **ERCC5** ] , XPFand CSB mRNAs were moderately correlated r=0.2 0.7.  Ref: 11056291 Mutat Res, 2000
- Here we show that with a highly defined system of purified repair factors, DDB can greatly stimulate the excision reaction reconstituted with XPA, RPA, XPC.HR23B, TFIIH  [ **ERCC3** ] , XPF.ERCC1 and XPG  [ **ERCC5** ] , up to 17 fold for CPDs and approximately 2 fold for 6 4 photoproducts 6 4PPs, indicating that no additional factor is required for the stimulation by DDB.  Ref: 11705987 J Biol Chem, 2002
- The dual incisions are accomplished by sequential and partly overlapping actions of six repair factors, RPA, XPA, XPC, TFIIH  [ **ERCC3** ] , XPG  [ **ERCC5** ] , and XPF.ERCC1.  Ref: 10373492 J Biol Chem, 1999
- Such genes include ERCC1, XPA, XPB  [ **ERCC3** ] , XPD, XPF, and XPG  [ **ERCC5** ] .  Ref: 10891549 Int J Oncol, 2000
- However, cells deficient in the other NER genes, XPD, XPB  [ **ERCC3** ]  and XPG  [ **ERCC5** ] , all have the same ratio of exchanges to breaks as wild type.  Ref: 12509251 DNA Repair Amst , 2002
- Additionally we have examined interactions between TFIIH  [ **ERCC3** ]  components, the human NER protein XPG  [ **ERCC5** ] , and the CSB protein which is implicated in Cockayne syndrome CS.  Ref: 8652557 Biochemistry, 1996
- these can be further subdivided into mutants which are defective in nucleotide excision repair alone UV5 ERCC2, UV24  **ERCC3** , UV135  **ERCC5**  and UV61 ERCC6 compared to those which have an associated defect in the distinct but overlapping pathway for the repair of DNA crosslinks UV20 ERCC1 and UV41 ERCC4.  Ref: 9339946 Radiat Res, 1997
- Loss of heterozygosity LOH analysis, using the microdissected tissues, for the XPA, XPB  [ **ERCC3** ] , XPC, XPD, XPE, XPF, XPG  [ **ERCC5** ]  and the transcription coupled repair factor, Cockayne syndrome B CSB revealed that NER factors were abnormal in 30.0% 3 10 cases of oral squamous cell carcinomas.  Ref: 11496330 Oncol Rep< MedlineTA>Oncol Rep, 1997
- Core NER enzymes XPA, XPF, XPG  [ **ERCC5** ] , and TFIIH  [ **ERCC3** ]  are essential for recovery.  Ref: 12531012 DNA Repair Amst , 2002
- For the bulk of mammalian DNA, the core protein factors needed for damage recognition and incision during nucleotide excision repair NER are the XPA protein, the heterotrimeric RPA protein, the 6 to 9 subunit TFIIH  [ **ERCC3** ] , the XPC hHR23B complex, the XPG  [ **ERCC5** ]  nuclease, and the ERCC1 XPF nuclease.  Ref: 10214908 Biochimie< MedlineTA>Biochimie, 2002
- XPA and RPA, once forming a complex at the damage site, would likely work with TFIIH  [ **ERCC3** ] , XPG  [ **ERCC5** ] , and ERCC1 XPF for dual incision.  Ref: 12486030 J Biol Chem, 2003
- In a pilot case control study, we measured the relative expression levels of five NER genes ERCC1, XPB  [ **ERCC3** ]   **ERCC3** , XPG  [ **ERCC5** ]   **ERCC5** , CSB ERCC6 and XPC ERCC, excision repair cross complementing.  Ref: 10910954 Carcinogenesis, 2000
- In multivariate logistic regression analysis, low expression of ERCC1, XPB  [ **ERCC3** ]   **ERCC3** , XPG  [ **ERCC5** ]   **ERCC5** , and CSB ERCC6 was associated with a statistically significant increased risk for SCCHN.  Ref: 11900225 Cancer, 2002
- mRNA levels of XPA, XPB  [ **ERCC3** ] , XPC, XPD, XPF, XPG  [ **ERCC5** ] , CSB and ERCC1 in primary lymphocytes from 33 individuals were quantified by dot blots and normalized to beta actin.  Ref: 11056291 Mutat Res, 2000
- Another class of patients with CS symptoms have mutations in the XPB  [ **ERCC3** ] , XPD, or XPG  [ **ERCC5** ]  genes, which result in UV hypersensitivity as well as defective global NER.  Ref: 11443545 Am J Hum Genet, 2001
- Finally, damage DNA excision reaction on SV40 minichromosomes was reconstituted with six highly purified NER factors, XPA, XPC HR23B, XPF ERCC1, XPG  [ **ERCC5** ] , RPA and TFIIH  [ **ERCC3** ] , as those essential for the reaction with naked DNA.  Ref: 10725665 Mutat Res, 2000
- Loss of heterozygosity LOH analysis for the XP, XPA, XPB  [ **ERCC3** ] , XPC, XPD, XPE, XPF, XPG  [ **ERCC5** ]  and the transcription coupled repair factor, Cockayne syndrome B CSB revealed that NER factors were abnormal in 62.1 % of ovarian tumors 18 2 6.7% of colon 2 12 and 22.2% lung 2 9 carcinomas.  Ref: 11689286 Cancer Lett, 2001
- The proteins known to be involved in the first steps of the NER reaction damage recognition and incision excision are heterotrimeric RPA, XPA, the 6 to 9 subunit TFIIH  [ **ERCC3** ] , XPC hHR23B, XPG  [ **ERCC5** ] , and ERCC1 XPF.  Ref: 10526214 Mutat Res, 1999
- Transcription coupled repair of 8 oxoguanine requirement for XPG  [ **ERCC5** ] , TFIIH  [ **ERCC3** ] , and CSB and implications for Cockayne syndrome.Analysis of transcription coupled repair of oxidative lesions here reveals strand specific removal of 8 oxo guanine and thymine glycol both in normal human cells and xeroderma pigmentosum cells defective in nucleotide excision repair.  Ref: 10786832 Cell, 2000
